# Clinical impact of and microbiological risk factors for *qacA/B* positivity in ICU-acquired ST5-methicillin-resistant SCC*mec* type II *Staphylococcus aureus* bacteremia

**DOI:** 10.1038/s41598-022-15546-3

**Published:** 2022-07-06

**Authors:** Haein Kim, Sunghee Park, Hyeonji Seo, Hyemin Chung, Eun Sil Kim, Heungsup Sung, Mi-Na Kim, Seongman Bae, Jiwon Jung, Min Jae Kim, Sung-Han Kim, Sang-Oh Lee, Sang-Ho Choi, Yang Soo Kim, Yong Pil Chong

**Affiliations:** 1 Department of Infectious Diseases, Asan Medical Center, University of Ulsan College of Medicine, 88 Olympic-ro-43-gil, Songpa-gu, Seoul, 05505 South Korea; 2 Department of Laboratory Medicine, Asan Medical Center, University of Ulsan College of Medicine, Seoul, Republic of Korea; 3 Asan Institute for Life Sciences, Asan Medical Center, University of Ulsan College of Medicine, Seoul, Republic of Korea

**Keywords:** Bacterial infection, Antimicrobial resistance

## Abstract

Concern about resistance to chlorhexidine has increased due to the wide use of the latter. The impact of the *qacA/B* and *smr* chlorhexidine tolerance genes on the outcome of methicillin-resistant *Staphylococcus aureus* (MRSA) infections is unclear. We evaluated the prevalence and clinical impact of, and microbiological risk factors for, *qacA/B* tolerance in MRSA bacteremia. MRSA bacteremia that occurred more than two days after intensive care unit admission between January 2009 and December 2018 was identified from a prospective cohort of *S. aureus* bacteremia in a tertiary-care hospital from South Korea. A total of 183 MRSA blood isolates was identified, and the major genotype found was ST5-MRSA-II (87.4%). The prevalences of *qacA/B* and *smr* were 67.2% and 3.8%, respectively. *qacA/B*-positive isolates were predominantly ST5-MRSA-II (96.7% [119/123]), the dominant hospital clone. In a homogenous ST5-MRSA-II background, *qacA/B* positivity was independently associated with septic shock (aOR, 4.85), gentamicin resistance (aOR, 74.43), and non-t002 *spa* type (aOR, 74.12). *qacA/B* positivity was found to have decreased significantly in ST5-MRSA-II in association with a decline in *qacA/B*-positive t2460, despite the increasing use of chlorhexidine since 2010 (*P* < 0.001 for trend). Continuous surveillance of the *qac* genes, and molecular characterization of their plasmids, are needed to understand their role in MRSA epidemiology.

## Introduction

Methicillin-resistant *Staphylococcus aureus* (MRSA) is one of the most common causes of healthcare-associated infection. It leads to increased morbidity and mortality in critically ill patients in intensive care units (ICUs). In the United States, the rate of hospital-acquired *Staphylococcus aureus* bacteremia was 31% and had decreased by 1.9% annually from 1995 to 2015. However, the frequency of metastatic complications of *S. aureus* bacteremia has increased by 0.9% per year, and the overall mortality rate is still high at 26.5%^1^. In South Korea, the hospital-acquired *S. aureus* bacteremia rate was 56% and had decreased by 2.4% annually from 2008 to 2018, and the 90-day mortality rate was 27% in 2018. The proportion of MRSA in *S. aureus* bacteremia was 52%, with a decrease of 1.6% annually^2^.

Infection control strategies to minimize MRSA infection include education about hand washing, various MRSA bundles, and chlorhexidine bathing with or without topical nasal mupirocin application^3,4^. Chlorhexidine bathing is estimated to reduce MRSA colonization and bacteremia in the ICU by 41% and 36%, respectively^5^. While there is benefit from chlorhexidine bathing and nasal mupirocin application, concern persists about the potential emergence of increased resistance due to antiseptic exposure. The *qacA* and *qacB* genes encode multidrug efflux pumps associated with chlorhexidine tolerance^6,7^. Several studies have shown an increase in the proportion of these genes after the widespread use of chlorhexidine in hospitals^8–10^. In addition, the presence of the *qacA* or *qacB* (*qacA/B*) gene has been linked to resistance to gentamicin, clindamycin, and penicillin, as well as to a high vancomycin minimal inhibitory concentration (MIC)^11–13^.

Previous studies have shown an association between *qacA/B* positivity and specific MRSA genotypes, including ST5- and ST239-MRSA^6,7,14^. ST5-MRSA is a major hospital MRSA clone in South Korea, while ST72-MRSA is a community-associated MRSA^2^. Because ST5-MRSA is associated with higher mortality and more accessory gene regulator (*agr*) dysfunctional than ST72-MRSA^15,16^, clinical and microbiological risk factors for *qacA/B* positivity might be affected by the characteristics of specific genotypes. Thus, this study aimed to evaluate the prevalence and clinical impact of, and microbiological risk factors for, *qacA/B* positivity in MRSA bacteremia in a homogenous genotype background after the implementation of chlorhexidine use in a tertiary hospital from South Korea.

## Methods

### Study population and design

A retrospective analysis of a prospective observational cohort of patients with *S. aureus* bacteremia was conducted at the Asan Medical Center, a 2700-bed tertiary care hospital with 118 adult ICU beds, in Seoul, South Korea. All consecutive adult patients (≥ 16 years old) who had MRSA bacteremia that developed more than two days after ICU admission between January 2009 and December 2018 were identified, and only patients whose MRSA isolates were collected and available for laboratory tests were included in the study^17^. Only the initial episode of MRSA bacteremia in any given patient was included. Recurrence of *S. aureus* bacteremia more than three months from the first bacteremia episode was regarded as a new episode and included in the analysis.

To evaluate the clinical impact of *qacA/B* positivity in a single genotypic background, multilocus sequence typing (MLST) of the MRSA isolates was performed. The clinical, microbiological, and genotypic characteristics of *qacA/B*-positive MRSA isolates, and risk factors for *qacA/B* positivity, were analyzed. Treatment outcome of bacteremia was evaluated based on 14-day, 30-day, and 12-week all-cause mortality. This study was approved by the Asan Medical Center Institutional Review Board (IRB number: 2008-0274). The requirement for written informed consent was waived by the Asan Medical Center Institutional Review Board, given the observational nature of the study. All methods were performed in accordance with the relevant guidelines and regulations.

### Study definitions

The prognosis for the underlying disease was classified according to the system of McCabe and Jackson as rapidly fatal (when death was expected within a few months), ultimately fatal (when death was expected within 4 years), and nonfatal (when life expectancy was > 4 years)^18^. Charlson’s comorbidity index was used to measure comorbid burden^19^. The severity of bacteremia was measured by the Acute Physiology and Chronic Health Evaluation II (APACHE II) score and the Pitt bacteremia score^20^. Sites of infection causing MRSA bacteremia were defined according to the Centers for Disease Control and Prevention criteria^21^. Catheter-related infection was defined according to the Infectious Diseases Society of America guidelines^22^.

### Microbiological data and determination of genotypes

All *S. aureus* isolates were identified by standard methods. Antimicrobial susceptibilities were determined with a MicroScan (Dade Behring, West Sacramento, CA, USA) and the standard criteria of the Clinical and Laboratory Standards Institute (CLSI)^23^. Methicillin resistance was confirmed by detection of the *mecA* gene by PCR. Vancomycin minimal inhibitory concentrations (MICs) were determined by the vancomycin E-test (AB Biodisk, Piscataway, NJ, USA) on Mueller–Hinton agar. Heterogeneous vancomycin-intermediate *S. aureus* (hVISA) was detected by population analysis profiling, as previously described^24^.

Staphylococcal Cassette Chromosome *mec* (SCC*mec*) type, MLST, and *Staphylococcus* protein A (*spa*) typing, as well as *agr* genotyping, were performed as described elsewhere^25–28^. MLST allele numbers and sequence types (STs) are available in the MLST database (http://www.mlst.net). *agr* dysfunction was detected by δ-hemolysin production^29^.

Chlorhexidine MIC was determined by the broth microdilution test described by CLSI^30^. Chlorhexidine digluconate 20% aqueous solution (Sigma-Aldrich, St. Louis, MO) was used as the starting material. The bacterial inocula (5 × 10^5^ colony-forming unit/mL) were seeded to wells containing chlorhexidine from 0.125 to 128 mg/L and incubated at 35 ℃ for 18 h. *S. aureus* ATCC 29213 was used as a quality control strain in susceptibility testing. Mupirocin resistance (low and high level) was detected by the disk diffusion method^31^, and high-level mupirocin resistance was confirmed by PCR detection of the *mupA* gene. The *qacA/ qacB, smr*, and *mupA* genes were detected by PCR using primers described elsewhere^32^.

### Infection control measures

From January 2010, our ICUs implemented a central venous catheter bundle including skin cleaning with 2% chlorhexidine gluconate in 70% alcohol. Once-daily chlorhexidine bathing was performed on patients with MRSA infections or colonizations in the surgical ICU from August 2011 and in the medical ICU from April 2013. Chlorhexidine gluconate-impregnated film dressing was used in 2016. All ICUs performed chlorhexidine bathing from August 2018. Topical nasal mupirocin has not usually been used for decolonization in ICU patients.

### Statistical analysis

We analyzed categorical variables using the chi-square or Fisher’s exact test, and continuous variables using Student’s *t*-test or the Mann–Whitney *U* test. A univariate analysis of the risk factors for *qacA/B*-positive MRSA was performed. All variables with *P* values < 0.1 in the univariate analysis were included in a multiple logistic regression model to identify independent risk factors. These variables were examined for correlations before inclusion in the multivariate analysis. Categorical variables were analyzed by the chi*-*square test, and continuous variables over time were analyzed by linear regression for trend. All tests of significance were two-tailed, and *P* < 0.05 was considered statistically significant. All statistical analyses were performed with SPSS, version 24.0 K for Windows (SPSS Inc, Chicago, IL, USA) and MedCalc software (Mariakerke, Belgium).

## Results

### Prevalence of qacA/B in MRSA blood isolates

During the study period, 238 episodes of ICU-acquired MRSA bacteremia were identified. When the 183 episodes with available MRSA isolates were analyzed, the two major genotypes were ST5-MRSA-II-*agr* group II (87.4%), the hospital MRSA clone, and ST72-MRSA-IV-*agr* group I (7.1%), the community-associated MRSA clone in South Korea. Major *spa* types in ST5-MRSA-II were t2460 (59.4%), t9353 (14.4%), and t002 (8.1%), and those in ST72-MRSA-IV were t148 (23.7%), t664 (23.7%), and t324 (15.4%). The prevalences of the *qacA/B* and *smr* genes were 67.2% (123/183) and 3.8% (7/183), respectively. The *qacA/B*-positive MRSA isolates were predominantly ST5-MRSA-II (96.7% [119/123]), and all ST72-MRSA-IV isolates were *qacA/B*-negative. Because the clinical and microbiological characteristics of ST5- and ST72-MRSA are known to differ^15^, the impact of *qacA/B* positivity was analyzed in ST5-MRSA-II isolates only, to exclude any effect of clonal heterogeneity on *qacA/B* negativity.

### Clinical characteristics and treatment outcomes of patients with qacA/B-positive ST5-MRSA-II bacteremia

Of the 160 ST5-MRSA-II isolates, 119 (74.4%) were *qacA/B*-positive*.* The clinical characteristics and treatment outcomes of the 160 patients with ST5-MRSA-II bacteremia according to the presence of the *qacA/B* gene are presented in Table 1. Median age was 64 (interquartile range [IQR], 53 to 72 years), and 133 of the patients (70.6%) were male. While there were no significant differences in clinical characteristics and outcomes between the two groups, patients with *qacA/B*-positive ST5-MRSA-II had a tendency to have bacteremia earlier, to have undergone recent surgery, and suffer septic shock more than those with *qacA/B*-negative ST5-MRSA-II.

**Table 1:** Clinical characteristics and outcomes of patients with ST5-MRSA-II bacteremia according to *qacA/B* status.

Characteristic/outcome	ST5*-qacA/B* (+) (n = 119)	ST5*-qacA/B* (−) (n = 41)	All ST5 isolates (n = 160)	*P* value
Median age, years (IQR)	64 (53–72)	67 (57–73)	64 (53–72)	0.13
Male gender	82 (68.9)	31 (75.6)	113 (70.6)	0.42
MRSA colonization within 1 year before bacteremia	90/101 (89.1)	27/35 (77.1)	117/136 (86.0)	0.09
Prior antimicrobial use within 1 month	115 (96.6)	38 (92.7)	153 (95.6)	0.37
**Site of acquisition**				
Nosocomial	119 (100)	41 (100)	160 (100)	> 0.99
Length of hospital stay before bacteremia, days (IQR)	22 (12–42)	24 (14–80)	23 (12–44)	0.08
Length of ICU stay before bacteremia, days (IQR)	11 (5–25)	16 (7–56)	11 (5–28)	0.07
**Underlying disease/condition**				
Solid tumor	44 (37.0)	15 (36.6)	59 (36.9)	0.96
Hematologic malignancy	1 (0.8)	0	1 (0.6)	> 0.99
Diabetes mellitus	31 (26.1)	7 (17.1)	38 (23.8)	0.24
Hemodialysis dependence	10 (8.4)	7 (17.1)	17 (10.6)	0.14
Liver cirrhosis	25 (21.0)	11 (26.8)	36 (22.5)	0.44
Solid organ transplantation	20 (16.8)	10 (24.4)	30 (18.8)	0.28
Neutropenia	2 (1.7)	1 (2.4)	3 (1.9)	> 0.99
Immunosuppressive treatment^a^	55 (46.2)	18 (43.9)	73 (45.6)	0.80
Recent chemotherapy	3 (2.5)	1 (2.4)	4 (2.5)	> 0.99
Recent surgery within 3 months	65 (54.6)	15 (36.6)	80 (50.0)	0.05
**McCabe and Jackson criteria**				
Ultimately or rapidly fatal disease	65 (54.6)	20 (48.8)	85 (53.1)	0.52
Charlson comorbidity index, median (IQR)	3 (2–5)	3 (2–5)	3 (2–5)	0.45
APACHE II score, median (IQR)	21 (16–27)	22 (18–29)	21 (17–28)	0.26
Pitt bacteremia score, median (IQR)	4 (2–6)	4 (2–5)	4 (2–5)	0.16
Septic shock	34 (28.6)	6 (14.6)	40 (25.0)	0.08
Central venous catheter	113 (95.0)	38 (92.7)	151 (94.4)	0.70
Prosthetic device^b^	17 (14.3)	3 (7.3)	20 (12.5)	0.25
**Site of infection**				
Catheter-related bacteremia	72 (60.5)	23 (56.1)	95 (59.4)	0.62
Pneumonia	22 (18.5)	10 (24.4)	32 (20.0)	0.42
Infective endocarditis	1 (0.8)	0	1 (0.6)	> 0.99
Postoperative wound infection	11 (9.2)	4 (9.8)	15 (9.4)	> 0.99
Primary bacteremia	11 (9.2)	3 (7.3)	14 (8.8)	> 0.99
Others	2 (1.7)	1 (2.4)	3 (1.9)	> 0.99
**Eradicable focus**	77 (64.7)	26 (63.4)	103 (64.4)	0.88
Removal of eradicable focus/eradicable focus	72/77 (93.5)	25/26 (96.2)	97/103 (94.2)	> 0.99
Persistent bacteremia (≥ 3 days)	35 (29.4)	16 (39.0)	51 (31.9)	0.26
Persistent bacteremia (≥ 7 days)	15 (12.6)	8 (19.5)	23 (14.4)	0.28
Recurrence within 3 months	9 (7.6)	3 (7.3)	12 (7.5)	> 0.99
14-day mortality	19 (16.0)	7 (17.1)	26 (16.3)	0.84
30-day mortality	43 (36.1)	16 (39.0)	59 (36.9)	0.74
12-week mortality	58 (48.7)	20 (48.8)	78 (48.8)	0.99

Data are no. (%) of patients, unless otherwise indicated.

*ICU* intensive care unit, *IQR* interquartile range, *MRSA* methicillin resistant *Staphylococcus aureus*, *NA* not applicable, *SD* standard deviation.

^a^Immunosuppressive treatment includes immunosuppressant use (≥ 7 days), steroid use (≥ 14 days), or radiation therapy (within 1 month).

^b^Prosthetic devices include pacemaker, ICD defibrillator, prosthetic heart valves, vascular grafts, orthopedic devices, and major artery stent.

### Microbiological and genotypic characteristics of the qacA/B-positive ST5-MRSA-II isolates

The most common *spa* type of ST5-MRSA-II was t2460 (59.4%), followed by t9353 (14.4%) and t002 (8.1%). As shown in Table 2, t9353 was significantly associated with *qacA/B* positivity (18.5% vs 2.4%, *P* = 0.01), and t002 with *qacA/B* negativity (1.7% vs. 26.8%, *P* < 0.001). The *qacA/B*-positive isolates were more often resistant to fusidic acid (93.3% vs. 73.2%, *P* = 0.001) and gentamicin (95.8% vs. 43.9%, *P* < 0.001) than were the *qacA/B*-negative isolates. The association of gentamicin resistance with *qacA/B* positivity was pronounced in the t2460 isolates (100% [74/74] vs. 33.3% [7/21], *P* < 0.001). In addition, the *qacA*/*B*-positive isolates had significantly higher chlorhexidine MICs than the negative ones (*P* < 0.001) (Table 2 and Supplemental Fig. S1). The chlorhexidine MIC_50_ and MIC_90_ values were 4 mg/L and 4 mg/L in *qacA/B*-positive isolates, whereas those were 1 mg/L and 2 mg/L, respectively, in *qacA/B*-negative isolates. However, mupirocin resistance and presence of the *mupA* gene were not associated with *qacA/B* positivity. Presence of the *smr* gene, another chlorhexidine tolerance gene, was significantly associated with *qacA/B* negativity (*P* = 0.04).

**Table 2:** Microbiological and genotypic characteristics of ST5-MRSA-II blood isolates according to *qacA/B* status.

Characteristic	ST5*-qacA/B* (+) (n = 119)	ST5*-qacA/B* (−) (n = 41)	All ST5 isolates (n = 160)	*P* value
**Vancomycin MIC (E test)**				
MIC, median^a^ (range), mg/L	1.5 (1–2)	1.5 (1–2)	1.5 (1–2)	0.43
MIC_90,_ mg/L	2	2	2	NA
≤ 1 mg/L	35 (29.4)	13 (31.7)	48 (30.0)	0.78
1.5 mg/L	50 (42.0)	15 (36.6)	65 (40.6)	0.54
≥ 2 mg/L	34 (28.6)	13 (31.7)	47 (29.4)	0.70
hVISA phenotype	46 (38.7)	15 (36.6)	61 (38.1)	0.81
***spa* type**				
t2460	74 (62.2)	21 (51.2)	95 (59.4)	0.22
t9353	22 (18.5)	1 (2.4)	23 (14.4)	0.01
t002	2 (1.7)	11 (26.8)	13 (8.1)	< 0.001
t148	1 (0.8)	0 (0)	1 (0.6)	> 0.99
t264	3 (2.5)	1 (2.4)	4 (2.5)	> 0.99
t9363	3 (2.5)	1 (2.4)	4 (2.5)	> 0.99
Others	14 (11.8)	6 (14.6)	20 (12.5)	0.63
*agr* dysfunction	118 (99.2)	37 (90.2)	155 (96.9)	0.02
**Antimicrobial resistance**				
Clindamycin	117 (98.3)	41 (100)	158 (98.8)	> 0.99
Ciprofloxacin	118 (99.2)	41 (100)	159 (99.4)	> 0.99
Erythromycin	119 (100)	40 (97.6)	159 (99.4)	0.26
Fusidic acid	111 (93.3)	30 (73.2)	141 (88.1)	0.001
Gentamicin	114 (95.8)	18 (43.9)	132 (82.5)	< 0.001
Rifampin	16 (13.4)	4 (9.8)	20 (12.5)	0.84
Trimethoprim/sulfamethoxazole	2 (1.7)	1 (2.4)	3 (1.9)	> 0.99
Tetracycline	103 (86.6)	33 (80.5)	136 (85.0)	0.35
**Chlorhexidine MIC**				
MIC, median^a^ (range), mg/L	4 (2–4)	1 (1–2)	3 (2–4)	< 0.001
MIC_90_, mg/L	4	2	4	NA
1 mg/L	2 (1.7)	21 (51.2)	23 (14.4)	< 0.001
2 mg/L	39 (32.8)	18 (43.9)	57 (35.6)	0.20
4 mg/L	78 (65.5)	2 (4.9)	80 (50.0)	< 0.001
**Mupirocin resistance**				
Low-level resistance	41 (34.5)	14 (34.1)	55 (34.4)	0.97
High-level resistance	4 (3.4)	3 (7.3)	7 (4.4)	0.37
**Presence of resistance gene**				
*smr*	2 (1.7)	4 (9.8)	6 (3.8)	0.04
*mupA*	4 (3.4)	4 (9.8)	8 (5.0)	0.21

Data are no. (%) of patients, unless otherwise indicated.

*hVISA* heterogeneous vancomycin-intermediate *Staphylococcus aureus*, *MIC* minimal inhibitory concentration, *MRSA* methicillin resistant *Staphylococcus aureus*, *NA* not applicable, *SD* standard deviation.

^a^Median MIC means MIC_50_.

**Figure 1:**
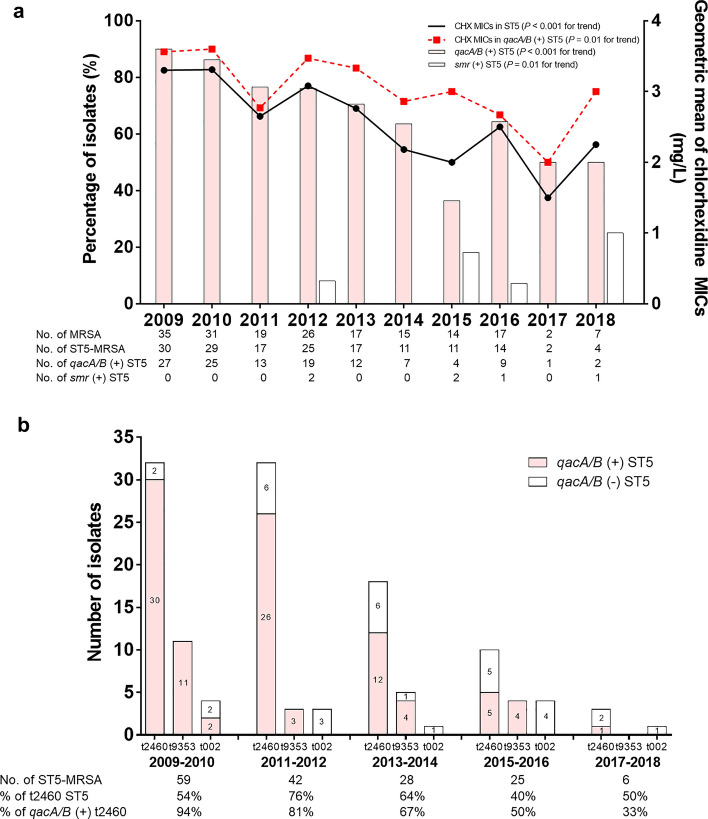
Changes in microbiological and genotypic characteristics of ST5-MRSA-II blood isolates. (**a**) The proportion of *qacA/B*-positive isolates in ST5-MRSA-II and geometric means of chlorhexidine (CHX) MICs decreased over time (*P* < 0.001 for trend for both). (**b**) Changes in *spa* types. The proportion of *qacA/B*-positivity in t2460 decreased over time (*P* < 0.001 for trend).

The proportion of *qacA/B*-positive isolates decreased from 88.1 to 50% over time (*P* < 0.001 for trend) (Fig. 1a), mainly due to the lowered *qacA/B*-positive rate in t2460 (*P* < 0.001 for trend) (Fig. 1b).

### Risk factors for qacA/B-positive ST5-MRSA-II isolates

Univariate variables with *P* values of less than 0.1 in Tables 1 and 2 were included in a logistic regression model to identify independent risk factors for *qacA*/*B*-positive MRSA. Because the t9353 *spa* type and fusidic acid resistance were highly correlated with gentamicin resistance, we retained gentamicin resistance only. Also, we included length of ICU stay before bacteremia rather than length of hospital stay before bacteremia because the former is a more clinically meaningful variable in ICU-acquired MRSA bacteremia. Thus, included in the multivariate regression modeling were length of ICU stay before bacteremia (per 10 days), recent surgery, septic shock, gentamicin resistance, non-t002 *spa* type, and *agr* dysfunction. The independent risk factors associated with *qacA/B* positivity were found to be septic shock (adjusted odds ratio [aOR], 4.85; 95% confidence interval [CI], 1.09 to 21.51), gentamicin resistance (aOR, 74.43; 95% CI, 19.19 to 288.67), and non-t002 *spa* type (aOR, 74.12; 95% CI, 10.99 to 499.60) (Table 3).

**Table 3:** Univariate and multivariate analyses of risk factors for presence of *qacA/B* in ST5-MRSA-II blood isolates.

Variable	Univariate analysis		Multivariate analysis^a^	
	OR (95% CI)	*P* value	aOR (95% CI)	*P* value
Length of ICU stay before bacteremia, per 10 days	0.94 (0.87–1.01)	0.10		
Recent surgery within 3 months	2.09 (1.01–4.33)	0.05		
Septic shock	2.33 (0.90–6.05)	0.08	4.85 (1.09–21.51)	0.04
Gentamicin resistance	29.13 (9.82–86.43)	< 0.001	74.43 (19.19–288.67)	< 0.001
non-t002 *spa* type	21.45 (4.51–101.97)	< 0.001	74.12 (10.99–499.60)	< 0.001
*agr* dysfunction	12.76 (1.38–117.72)	0.03		

Data are no. (%) of patients, unless otherwise indicated.

*OR* odds ratio, *CI* confidence interval.

^a^This model fits the data well in terms of discrimination (C-statistic = 0.91) and calibration (Hosmer–Lemeshow goodness-of-fit statistic = 8.62; *P* = 0.28).

## Discussion

In this study we found that the chlorhexidine tolerance genes *qacA/B* and *smr* were mainly present in the specific hospital MRSA clone, ST5-MRSA-II, after the implementation of chlorhexidine use in a tertiary hospital from South Korea. The *qacA/B* gene was independently associated with septic shock, gentamicin resistance, and non-t002 *spa* type (especially *spa* type t9353) in a homogenous ST5-MRSA-II background. This implies that the *qacA/B* coexists well with the t9353 *spa* type but not the t002 *spa* type. During the study period, *qacA/B* positivity decreased significantly in ST5-MRSA-II mainly due to a reduction in *qacA/B*-positive t2460 isolates despite the increasing use of chlorhexidine in this hospital since 2010.

Previous clinical and microbiological studies of *qacA/B* and *smr* had been performed on strains with heterogeneous genotypic and clinical backgrounds, including both community and healthcare *S. aureus* strains^6,13,33,34^. Therefore, the proportion of *qacA/B*-positive isolates in the present study, which included only ICU-acquired ST5-MRSA-II bacteremia, was higher (74.4%) than in previous studies. Interestingly, the proportion of *smr*-positive isolates was very low (3.8%), in contrast with previous studies^13,33,34^. In another study conducted in South Korea^6^, the proportion of *smr*-positive MRSA was also low (2.8%), and *qacA/B* positivity was closely associated with ST5-MRSA, the dominant and generally *agr* dysfunctional hospital clone^16^. ST72-MRSA, a community-associated MRSA clone in South Korea that is usually *agr* functional^16^, was also significantly associated with *qacA/B* negativity in that study. Therefore, factors that were found to be independently associated with *qacA/B* positivity in that study, such as *agr* dysfunction, nosocomial infection, and previous antibiotic use, could have been reflections of the characteristics of the specific MLST, ST5-MRSA, because all MRSA genotypes were included. The clonal association of *qacA/B* with hospital MRSA clones such as ST239, ST5, and ST241 have been found in Asia^14,35^. Therefore, the clonal association between *qacA/B* and ST5-MRSA-II isolates in present study could be influenced by the dominant regional hospital MRSA clone in South Korea.

In contrast to other studies that showed the increased proportion of *qacA/B* positivity after the widespread use of chlorhexidine in hospitals^8–10^, the present study revealed that *qacA/B* positivity in ST5-MRSA-II decreased over time. This finding could be influenced by the decreased proportion of *qacA/B*-positive rate in t2460, which was main *spa* type in ST5-MRSA-II.

Several studies have shown that healthcare exposure and underlying medical condition are independently associated with *qacA/B* positivity in *S. aureus* isolates^6,13,34^. In the present study, we found that septic shock, gentamicin resistance, and fusidic acid resistance were also closely associated with *qacA/B* positivity in nosocomial ST5-MRSA-II blood isolates in a homogenous MRSA genotypic background. The *qacA/B* genes are usually carried by large plasmids that encode efflux pumps, and some of these plasmids contain other antibiotic resistance genes^11,12,36^. This fact should influence the likelihood of co-resistance to other antimicrobials such as gentamicin and fusidic acid. In addition, the presence of virulence genes on certain *qac* plasmids could affect the severity of the illness of patients with *qacA/B*-positive MRSA bacteremia^37^. Further detailed analysis of our *qac* plasmids would clarify these associations.

Interestingly, the non-t002 *spa* type was significantly associated with *qacA/B* positivity in ST5-MRSA-II isolates. t2460 and t9353 are the major *spa* types of MRSA in South Korea^6,38^. While a previous study showed that t2460 was associated with the *qacA/B* genes in MRSA strains as a whole^6^, we found that only t9353 was strongly associated with *qacA/B* positivity in a homogenous ST5-MRSA-II background. As shown in Fig. 1b, there has been no outbreak of *qacA/B*-positive t9353, and the proportion of *qacA/B*-positivity in t2460 strains has decreased over time (*P* < 0.001 for trend). Potential hypotheses for such findings are that t9353 *spa* type might maintain the *qacA/B* gene efficiently, and t2460 may have a tendency to lose a plasmid carrying *qacA/B*.

This study has several limitations. First, it was conducted in a single tertiary-care hospital and included exclusively MRSA bacteremia occurring in ICUs. Hence, further studies are needed to confirm our results in other hospital settings. Second, because almost all the patients had a recent history of antibiotic use, it was difficult to determine whether antibiotic use exerts selective pressure for chlorhexidine tolerance. Third, despite the proportion of available MRSA blood isolates tested was high (183/238, 77%), these results may not reflect the true prevalence of the genotypes found. Therefore, caution is needed in generalizing the results to other situations.

In conclusion, the *qacA/B* and *smr* antiseptic tolerance genes were mainly encountered in ST5-MRSA-II, the dominant hospital MRSA clone, after the implementation of chlorhexidine use in a tertiary hospital from South Korea since 2010. Septic shock, gentamicin resistance, and non-t002 *spa* type were independent predictors of the presence of *qacA/B* in ST5-MRSA-II blood isolates. Continuous surveillance for the *qac* genes and molecular characterization of the corresponding plasmids are needed to understand the role of these genes in MRSA epidemiology.

## Supplementary Information

Supplementary Information.

## Data Availability

All data generated or analyzed during this study are included in this published article and its supplementary information files. The genotypic data have been deposited to the figshare database at https://doi.org/10.6084/m9.figshare.19114976.v2.
