# Supplementary material for: Clinical impact of and microbiological risk factors for qacA/B positivity in ICU-acquired ST5-methicillin-resistant SCCmec type II Staphylococcus aureus bacteremia
Source: Sci Rep. 2022 Jul 6;12:11413. doi: 10.1038/s41598-022-15546-3 (PMC9259651; doi:10.1038/s41598-022-15546-3)
Supplement: Supplementary file 1 — Supplementary Information. [file 41598_2022_15546_MOESM1_ESM.docx]

**Online Supplementary Data**

**Clinical impact of and microbiological risk factors for *qacA/B* positivity in ICU-acquired ST5-methicillin-resistant SCC*mec* type II *Staphylococcus aureus* bacteremia**

Haein Kim,^1^ Sunghee Park,^1^ Hyeonji Seo,^1^ Hyemin Chung,^1^ Eun Sil Kim,^1,3^ Heungsup Sung,^2^ Mi-Na Kim,^2^ Seongman Bae,^1^ Jiwon Jung,^1^ Min Jae Kim,^1^ Sung-Han Kim,^1^ Sang-Oh Lee,^1^ Sang-Ho Choi,^1^ Yang Soo Kim,^1^ Yong Pil Chong^1*^

^1^Department of Infectious Diseases, and ^2^Department of Laboratory Medicine, Asan Medical Center, University of Ulsan College of Medicine, Seoul, Republic of Korea

^3^Asan Institute for Life Sciences, Asan Medical Center, University of Ulsan College of Medicine, Seoul, Republic of Korea


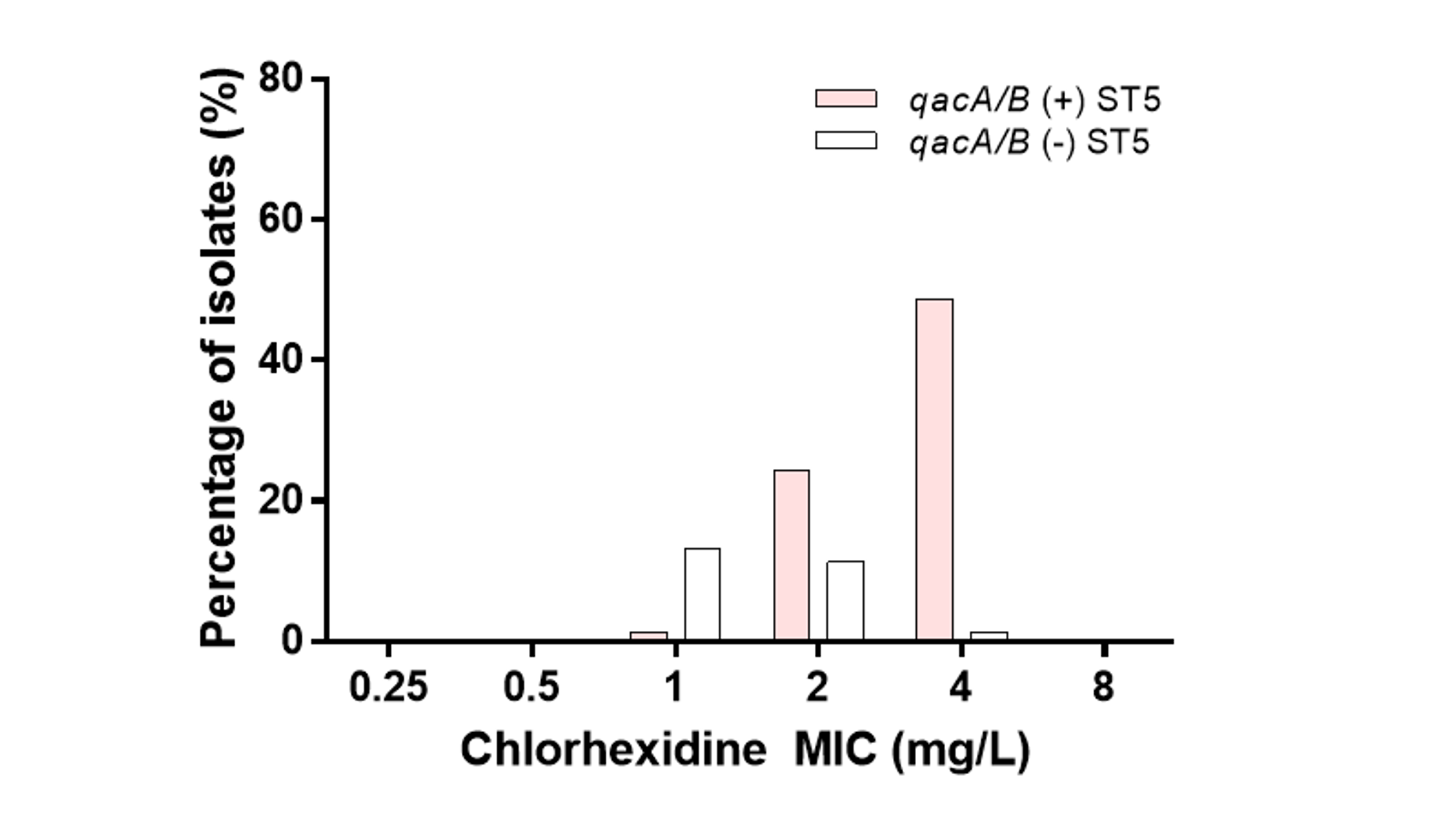
Supplemental Figure 1. Distribution of chlorhexidine MICs in ST5-MRSA-II blood isolates according to *qacA/B* status.
